# Engineered tissue geometry and Plakophilin-2 regulate electrophysiology of human iPSC-derived cardiomyocytes

**DOI:** 10.1063/5.0160677

**Published:** 2024-03-11

**Authors:** Daniel W. Simmons, Ganesh Malayath, David R. Schuftan, Jingxuan Guo, Kasoorelope Oguntuyo, Ghiska Ramahdita, Yuwen Sun, Samuel D. Jordan, Mary K. Munsell, Brennan Kandalaft, Missy Pear, Stacey L. Rentschler, Nathaniel Huebsch

**Affiliations:** 1 Department of Biomedical Engineering, Washington University in St. Louis McKelvey School of Engineering, St. Louis, Missouri 63130, USA; 2 Department of Mechanical Engineering and Materials Science, Washington University in St. Louis McKelvey School of Engineering, St. Louis, Missouri 63130, USA; 3 Department of Medicine, Cardiovascular Division, Washington University School of Medicine, St. Louis, Missouri 63110, USA

## Abstract

Engineered heart tissues have been created to study cardiac biology and disease in a setting that more closely mimics *in vivo* heart muscle than 2D monolayer culture. Previously published studies suggest that geometrically anisotropic micro-environments are crucial for inducing “*in vivo* like” physiology from immature cardiomyocytes. We hypothesized that the degree of cardiomyocyte alignment and prestress within engineered tissues is regulated by tissue geometry and, subsequently, drives electrophysiological development. Thus, we studied the effects of tissue geometry on electrophysiology of micro-heart muscle arrays (*μ*HM) engineered from human induced pluripotent stem cells (iPSCs). Elongated tissue geometries elicited cardiomyocyte shape and electrophysiology changes led to adaptations that yielded increased calcium intake during each contraction cycle. Strikingly, pharmacologic studies revealed that a threshold of prestress and/or cellular alignment is required for sodium channel function, whereas L-type calcium and rapidly rectifying potassium channels were largely insensitive to these changes. Concurrently, tissue elongation upregulated sodium channel (Na_V_1.5) and gap junction (Connexin 43, Cx43) protein expression. Based on these observations, we leveraged elongated *μ*HM to study the impact of loss-of-function mutation in Plakophilin 2 (PKP2), a desmosome protein implicated in arrhythmogenic disease. Within *μ*HM, PKP2 knockout cardiomyocytes had cellular morphology similar to what was observed in isogenic controls. However, PKP2^−/−^ tissues exhibited lower conduction velocity and no functional sodium current. PKP2 knockout *μ*HM exhibited geometrically linked upregulation of sodium channel but not Cx43, suggesting that post-translational mechanisms, including a lack of ion channel-gap junction communication, may underlie the lower conduction velocity observed in tissues harboring this genetic defect. Altogether, these observations demonstrate that simple, scalable micro-tissue systems can provide the physiologic stresses necessary to induce electrical remodeling of iPS-CM to enable studies on the electrophysiologic consequences of disease-associated genomic variants.

## INTRODUCTION

Human induced pluripotent stem cell-derived cardiomyocytes (iPS-CMs) are promising alternatives to current high-throughput *in vitro* assays for predicting cardiotoxic effects of drugs, and studies on patient-derived iPS-CM have demonstrated correlations between *in vitro* results and clinical incidence of arrhythmias.^1^ However, iPS-CMs have significantly different electrophysiology than their postnatal counterparts.^2^ iPS-CMs have a high spontaneous beating rate, inefficient calcium handling, and altered sodium and potassium channel function, which largely persist even over long-term (up to 12 months) culture.^3,4^ These changes cause monolayer iPS-CM to exhibit both false-positive and false-negative readouts for drug induced toxicity and pro-arrhythmia.^5–8^ These inconsistencies are likely due to deficits in ion channel expression and/or function.^9^

In order to overcome the limitations of monolayer iPS-CM, a variety of engineered heart tissues have been created.^10–13^ Tissue environments provide necessary cues such as cell alignment and increased mechanical loading to mature cells.^14–22^ Culturing iPS-CM within these “*in vivo*-like” formats has led to a variety of positive outcomes, including increased expression of contractile proteins, higher conduction velocity, and global changes in electrophysiology. Importantly, the resulting tissues can more accurately predict the effects of pro- and anti-arrhythmic drugs compared to monolayers, presumably through electrical remodeling that yields physiologic ion channel expression and/or activity.^14–16,20–24^

Interestingly, different engineered tissue systems elicit differing levels of induced electrical remodeling, even without additional biophysical (e.g., pacing^20^) or biochemical (e.g., fatty acids^25,26^) stimuli. It remains unknown what common factor(s) these tissues contain that induce cardiomyocyte maturation, making it difficult to compare systems or determine how to further improve them.

Mechanical loading plays critical roles in regulating heart and cardiomyocyte physiology^11^ during development and disease progression.^27–30^ In particular, tissue geometry and prestress regulate cardiomyocyte morphology,^31^ multiple signaling pathways, ion channel expression, calcium handling, and overall animal mortality.^32^ Prestress is generally defined as a “resting” mechanical tension on structural objects (e.g., cables) that would cause extension where these objects to be released from boundary constraints.^33^ From a tissue engineering standpoint, prestress is an attractive candidate as a biophysical cue to apply to lab-grown cardiac tissue because simple continuum mechanics suggests that tissue prestress depends primarily on its geometry,^31^ which can be controlled in a simplistic, scalable manner without sophisticated devices (e.g., mechanical stretching systems). From a physiologic standpoint, the resting tension provided by prestress is analogous to preload that heart muscle experiences *in vivo*.^10,11^

Increased prestress on cardiomyocytes cultured in aligned 3D engineered tissue environments^14,16,20,21,23^ has been hypothesized as a key physical cue to induce maturation *in vitro*,^31^ analogous to the manner in which preload regulates *in vivo* heart formation and function.^28,34,35^ Here, we manipulated the geometry of iPS-CM based engineered heart tissues to control prestress. We leveraged the resulting, geometrically linked global remodeling of cardiomyocyte electrophysiology to study the electrical phenotype of loss-of-function mutations in Plakophilin (PKP2), a mechanical junction protein strongly associated with inherited arrhythmogenic disease.^36–38^

## RESULTS

### Tissue prestress regulates cardiomyocyte morphology

**FIG. 1.**
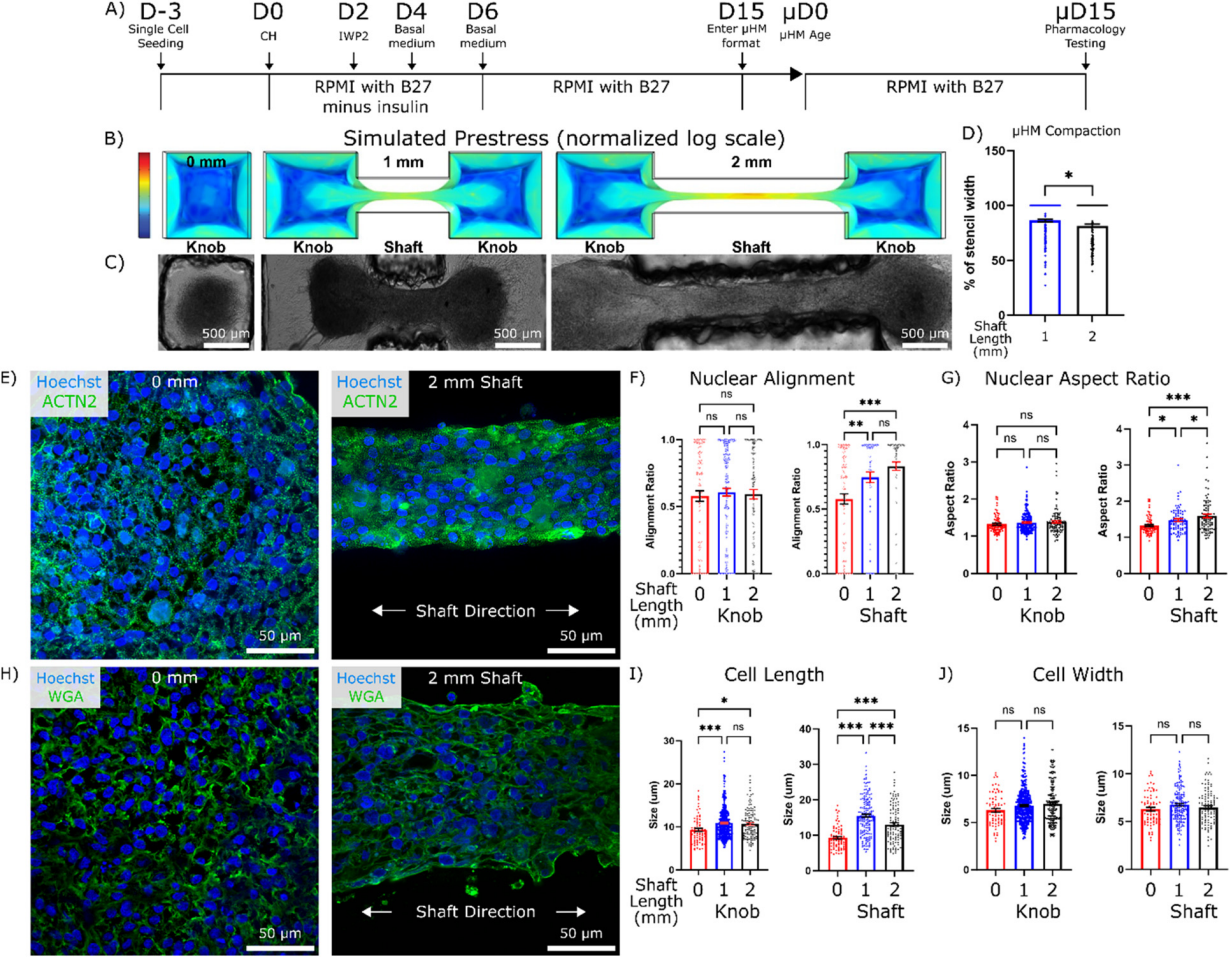
Prestress regulates cell morphology in micro-heart muscle (*μ*HM). (a) Timeline of small molecule-based differentiation of iPSC to cardiomyocytes, the subsequent seeding into the *μ*HM format, and the measurements of cell morphology, electrophysiology, and pharmacology experiments at the terminal time point. (b) COMSOL modeling images depicting increased tissue preload with increased shaft length. Color scale bar is log von Mises stress normalized to the 2 mm tissue. (c) Representative bright field images taken at *μ*HM day 15 (*μ*D15) showing grossly similar tissue morphology regardless of tissue size. (d) Quantification of *μ*HM shaft lateral compaction at *μ*D15 (*n* > 150 *μ*HM). (e) Representative confocal images of nuclei (Hoechst/blue) and sarcomeric α–actinin (green) of 0 and 2 mm tissues at *μ*D15. Quantification of the effects of tissue geometry on (f) nuclear alignment and (g) nuclear aspect ratio at *μ*D15, separated to compare 0 mm tissue to the knobs or shafts of 1- and 2-mm tissues (*n* > 70 nuclei from 6 to 8 *μ*HM per size). (h) Representative confocal images of nuclei (Hoechst/blue) and cell membrane (wheat germ agglutinin/green) of 0 and 2 mm tissues at *μ*D15. Quantification of the effects of tissue geometry on (i) cell length and (j) width at *μ*D15, separated to compare 0 mm tissue to the knobs or shafts of 1- and 2-mm tissues (n > 100 cells from 6 to 8 *μ*HM). (^*^: p < 0.05, ^**^: p < 0.005, ^***^: p < 0.0005, error bars: SEM).

To test the impact of cellular prestress and alignment on iPS-CM electrophysiology in 3D engineered heart tissue, we created micro-heart muscle arrays (*μ*HM) of various geometries. Simple continuum mechanics models of tissue compaction, which consider isotropic shrinkage of a hyperelastic solid against anisotropic boundary conditions, predict a direct link between tissue geometry and prestress. Specifically, prior studies with *μ*HM demonstrate delamination of the tissues from substrata within the rectangular “shaft” region of *μ*HM but suggest strong adhesion to substrates in the square-shaped “knobs” on either end of the tissue.^14,23^ Like other engineered tissues,^16^ cells seeded into *μ*HM molds compact into a tissue that is smaller than the volume of the originally seeded cell suspension. Based on observations, a simple continuum mechanics finite element model was constructed, in which mass of seeded iPS-CM was modeled as a Neo-Hookean material which remained fixed to the substrate under each knob but otherwise was allowed to undergo 50% 3D-volumetric shrinkage. This finite element simulation predicted increased von Mises stress and first principal stress within the central “shaft” region of the *μ*HM [Fig. 1(b); Fig. S1]. This prediction is similar to what others, including Abilez and colleagues, have described elsewhere.^31^

To control *μ*HM geometry, we used hydrogel assisted stereolithographic elastomer prototyping (HASTE)^39^ to create dogbone-shaped molds from 3D-printed resin. Three designs were produced: the first being an extreme case of an isolated knob (0 mm shaft length) in which little-to-no prestress was predicted based on finite element modeling, and the second and third designs consisting of *μ*HM with a 1- or 2-mm shaft length, respectively. These three resulting molds were seeded with iPS-CM at day 15 of differentiation^40^ (D15/*μ*HM day 0) and cultured as 3D tissues for an additional 15 days [*μ*D15; Figs. 1(a) and 1(b)]. Gross tissue morphology was largely unaffected by tissue shaft length within dogbone-shaped designs, although *μ*HM with longer shafts exhibited slightly more lateral compaction [Figs. 1(c) and 1(d)].

Despite the overall similarities in gross morphology between *μ*HM with 1 vs 2 mm shaft lengths, staining for sarcomeric α-actinin and cell membrane in longitudinal cryosections cut near the vertical center of *μ*HM suggested that tissue geometry had marked effects on cardiomyocyte structure [Figs. 1(e) and 1(h)]. Consistent with computational model predictions of the distribution of prestress in compacted tissues [Fig. 1(b)], morphology changes were limited mostly to cells located in the shaft region of the tissue. Nuclear alignment and nuclear aspect ratio were consistent across iPS-CM from the 0 mm tissues and the knob regions of the two elongated tissues [Figs. 1(f) and 1(g)]. In contrast, cardiomyocytes within the shaft regions of 1 and 2 mm *μ*HM exhibited increased nuclear alignment compared to iPS-CM within 0 mm tissues [Fig. 1(f)]. Similar results were observed for nuclear aspect ratio, although this measurement did indicate an increase in nuclear elongation within cells in the shaft region in 2 mm over 1 mm *μ*HM, whereas analysis of nuclear alignment did not [Fig. 1(g)]. Cell length showed a statistical increase between cells from the 0 mm tissues and those in the knob regions of the two elongated tissues [Fig. 1(i)], although this difference was minimal (∼1.5 *μ*m). In contrast, we observed a marked change in cell length (∼4–6 *μ*m) when comparing the 0 mm tissues to the shaft regions of the elongated tissues. Interestingly, cell length in the 2 mm *μ*HM shaft was slightly less than in iPS-CM from the shaft region of 1 mm *μ*HM [Fig. 1(i)]. This may be due to the increased compaction of the 2 mm *μ*HM. Importantly, although cell length increased in cells within *μ*HM with elongated shafts, cell width was unchanged [Fig. 1(j)]. This is consistent with eccentric hypertrophy, which is observed *in vivo* with increased preload.^35^ Collectively, these results confirm that increasing *μ*HM shaft length induces increasing levels of tissue prestress, analogous to preload that cardiomyocytes would experience *in vivo*. In turn, this prestress directly modulates cardiomyocyte morphology and alignment.

### Action potential and calcium handling kinetics are modulated by tissue prestress

**FIG 2.**
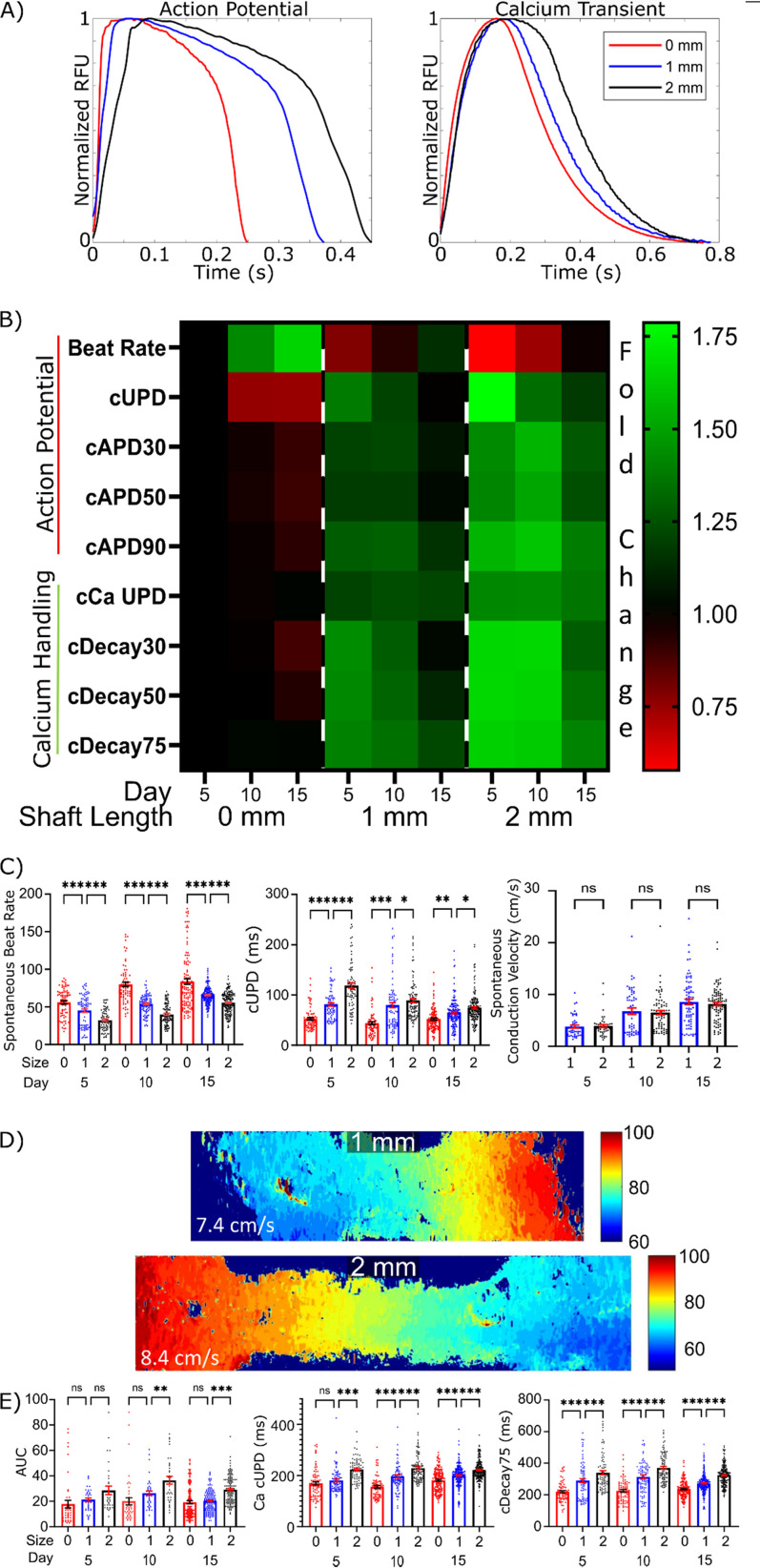
Tissue prestress regulates *μ*HM electro-physiology. (a) Representative action potential and calcium transient waveforms for 0, 1, and 2 mm *μ*HM at *μ*D15. (b) Electrophysiology heatmap of action potential and calcium handling dynamics for *μ*HM of shaft length 0, 1, and 2 mm long. Data are normalized to *μ*D5 0 mm data for given parameter. (c) Select data contained within the heatmap shown in panel B: spontaneous beat rate, beat-rate corrected action potential upstroke duration, and spontaneous conduction velocity. (d) Representative activation maps of 1 and 2 mm *μ*HM at *μ*D15. (e) Area under the curve of calcium transient waveforms, beat-rate corrected calcium transient upstroke duration, and beat-rate corrected calcium transient decay 75 for 0, 1, and 2 mm *μ*HM up to *μ*D15 (*n* > 100 *μ*HM, ^*^: p < 0.05, ^**^: p < 0.005, ^***^: p < 0.0005, error bars: *SEM*).

We next investigated potential effects of prestress on electrophysiology of spontaneously beating *μ*HM through optocardiography. Whole-tissue action potential and calcium transient waveforms displayed global remodeling as the *μ*HM shaft length increased [Fig. 2(a)]. Quantitative analysis of several key parameters describing the kinetics of action potentials and calcium transients (e.g., time to 90% action potential duration, APD_90_, and the time required for calcium transients to decay from peak to 25% of peak amplitude, τ_75_) confirmed the robustness of these prestress and/or cell alignment-induced global changes across a large number of *μ*HM amassed from multiple differentiation batches [Fig. 2(b), S4(d), and S4(e)]. Importantly, analysis of individual action potential and calcium transient kinetic parameters, such as cAPD_30_ and cDecay_30_, showed no measurable changes whether the entire tissue or a smaller sub-region was analyzed [Fig. S4(a)]. This suggests that *μ*HM electrophysiology is dominated by the regions of highest prestress within individual tissues. Consistent with a more mature phenotype,^2,26^ *μ*HM with longer shafts exhibited lower spontaneous beating rates [Fig. 2(c)]. Even after applying beat-rate correction using Fridericia's formula [Eq. (1)] to avoid over-estimating changes in action potential waveform kinetics [Figs. S4(b) and S4(c)],^41,42^ we observed that action potential upstroke duration (cUPD) and APD_90_ also increased substantially within *μ*HM with elongated geometries [Figs. 2(c) and S4(d)]. Beat-rate correction provides a conservative estimate of tissue geometry-induced changes in the 2 mm *μ*HM action potential waveform morphology: we observed trends toward lengthening of the action potential shaft length increased, and rate correction for spontaneous beat rates below 60 bpm [Figs. S4(b)–S4(d)] shortens cAPD_90_ estimates. This suggests against the possibility that observed changes in waveform morphology were simply a result of variations in beat rate.

Increases in action potential upstroke duration are most likely to reflect one of three possibilities: (1) decreased sodium current, which would suggest less mature tissues,^43^ (2) increased overall influx of ions into the cardiomyocytes during depolarization,^44^ or (3) increased gap-junction coupling between cells, which slows action potential and calcium upstroke timing in tissues.^45^ To address these distinct possibilities, we first analyzed the spontaneous conduction speed across *μ*HM, which is partially controlled by both sodium channel function and gap-junction coupling. Although distinctive in their overall action potential morphology, we observed no significant difference in conduction speed between 1- and 2-mm *μ*HM at any time point [Figs. 2(c) and 2(d)].

In contrast to conduction speed, which was consistent between *μ*HM with 1- and 2-mm shaft lengths, we observed marked differences in *μ*HM calcium handling as a function of shaft length. Both the upstroke duration and decay times of calcium transients increased with increasing tissue length [Fig. 2(e)]. Together, these changes led to an increase in the total amount of calcium influx into the cardiomyocytes, as measured by the area under the curve (AUC) of calcium transient waveforms [Fig. 2(e)]. Altogether, these results are most consistent with increases in total ion influx, rather than gross changes in gap junction functionality or sodium channel impairment, as the predominant mechanism for the observed increase in APD and upstroke timing. This change is consistent with previously published results associating increased Ca2+ influx with improved iPS-CM maturity^46,47^ and correlates with work revealing this increased influx directly leads to increased contractile strength.^48^

### Aligned tissue geometry is necessary for development of functional sodium currents

**FIG. 3.**
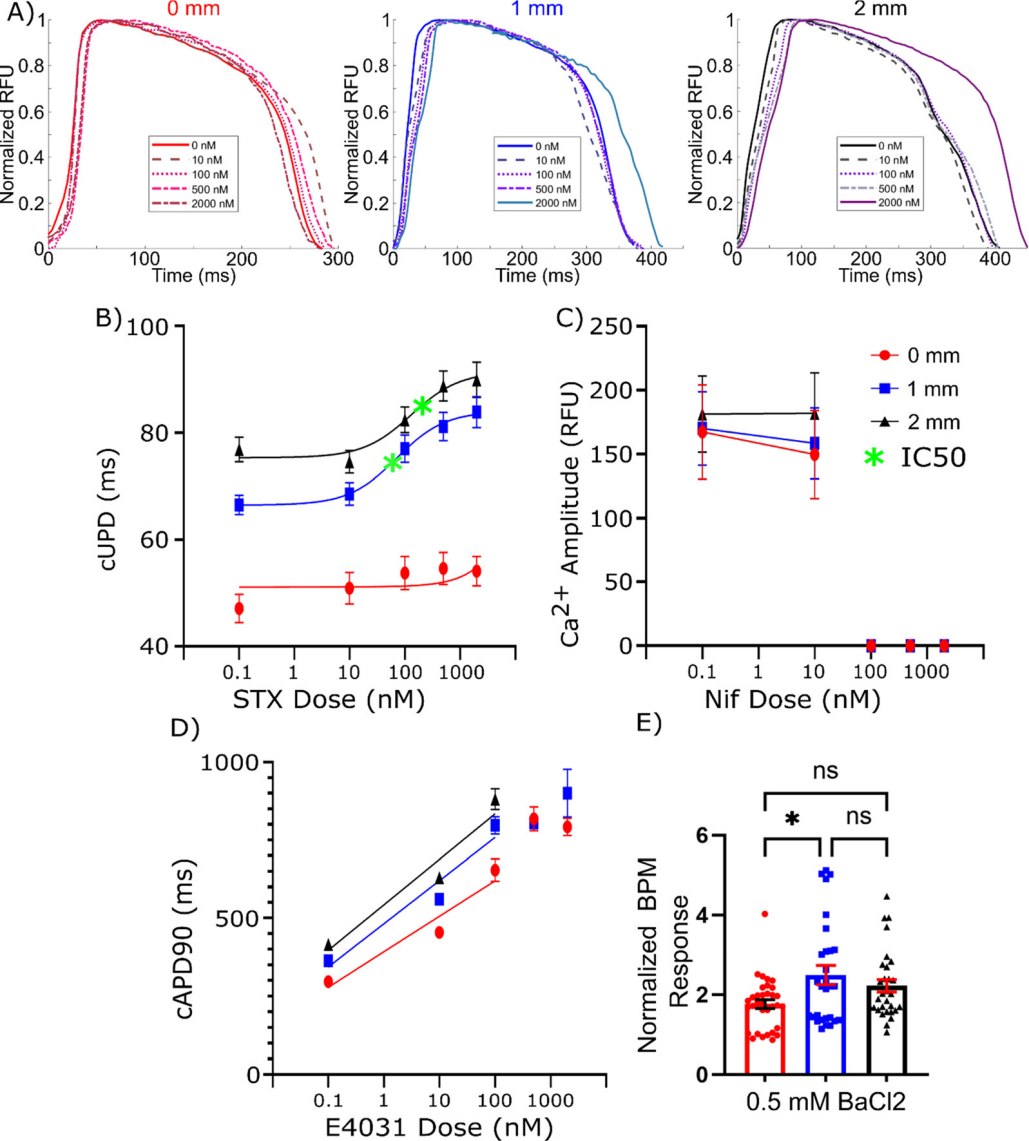
Pharmacologic inhibition of ion channels. (a) Representative action potential waveforms for 0, 1, and 2 mm *μ*HM after sequential saxitoxin doses. Response of *μ*HM to pharmacologic inhibition of ion channels. Effect of (b) saxitoxin on action potential upstroke duration, (c) nifedipine on calcium transient amplitude, (d) E4031 on ${\mathrm{cAPD}}_{90}$, and (e) 0.5 mM BaCl_2_ on spontaneous beat rate. (*n* = 20–30 *μ*HM, ^*^: p < 0.05, ^**^: p < 0.005, error bars: *SEM*).

We next used a series of probe drugs to block unique ion channels to determine what channels were most affected by tissue prestress and/or cell alignment to cause the observed electrical remodeling. We first assessed the sodium channel (Na_V_1.5) responsible for the action potential upstroke in the adult human cardiomyocyte and partially for the upstroke in matured iPS-CM,^49^ using saxitoxin. Strikingly, aligned tissues (*μ*HM with either 1- or 2-mm shaft length) exhibited a saxitoxin dose-response with increasing cUPD as *I_Na_* block increased, whereas tissues with no prestress or preferential alignment (0 mm) exhibited no such response [Figs. 3(a) and 3(b)]. These observations suggest that a threshold level of cellular alignment and/or prestress is necessary for Nav1.5 to make functional contributions to the action potential. Moreover, although the 1- and 2-mm tissues show similar *I_Na_* blockage curves in relation to cUPD, *μ*HM with 2 mm shaft length consistently maintained a higher cUPD. These observations strongly suggest that the increased upstroke time in *μ*HM with anisotropic, elongated geometry compared to the 0 mm *μ*HM [Figs. 2(a) and 2(b)] is due to the prevalence of increased *I_Na_*, as opposed to a Nav1.5 deficiency. A greater influx of Na^+^, and subsequently Ca^2+^, into cells would prolong the upstroke duration of the action potential and calcium transient,^44^ consistent with our findings (Fig. 2). This possibility was corroborated by a decrease in spontaneous conduction velocity in the presence of saxitoxin [Fig. S5(a)]. However, despite the increased sodium channel functionality in the 1- and 2-mm shaft-length tissues, high blocking doses of saxitoxin had minimal impacts on the spontaneous beat rate, action potential amplitude and duration in these tissues [Figs. S5(a) and S5(b)], highlighting that iPS-CMs in *μ*HM remain immature and the action potential upstroke is not completely dependent on *I_Na_* in these cells.

In contrast to observations linking prestress to functional *I_Na_*, blockade of either the *L*-type calcium current (*I_CaL_*) with nifedipine or the rapidly rectifying potassium current (*I_Kr_*) with E4031 revealed no marked changes in the calcium transient amplitude and cAPD90, respectively, based on tissue geometry [Figs. 3(c), 3(d), S6, and S7]. This suggests that the calcium transient shifts stemming from *μ*HM geometry changes [Figs. 2(a) and 2(b)] are most likely due to increases in *I_Na_* that allow more calcium to enter the cell, whereas calcium channels themselves are not significantly affected by tissue prestress and/or cellular alignment. Likewise, increases in APD_90_ with tissue prestress are unlikely to be caused by changes in *I_Kr_*, and instead are more likely to be caused by increased ion influx. Although elongated *μ*HM gain functional *I_Na_*, *I_CaL_* remains necessary for action potential initiation, as observed by the block of AP altogether at high dose of nifedipine (Fig. S6), and the finding that only a small component of conduction velocity is directly linked to *I_Na_* [Fig. S5(a)]. Additionally, moderate doses of 100 nM E4031 led to decreases in the action potential amplitude, with the effect increasing with E4031 concentration [Fig. S7(a)]. In numerous tissues, but not all, these higher doses also led to a complete cessation of beating. These effects are likely due to higher doses of E4031 blocking *I_Kr_* to such a degree that the cardiomyocytes are unable to adequately return to their baseline resting membrane potential after depolarization occurs with each spontaneous beat.

We next used barium chloride as a probe to address functional changes to *I_K_*_1_, which regulates resting membrane potential. The protein encoding *I_K_*_1_, Kir2.1, forms complexes with Na_V_1.5 in cardiomyocytes that are important for the trafficking of both proteins.^50,51^ In contrast to our observations for STX treatment, which suggested selective upregulation of *I_Na_* in 1 and 2 mm shaft length *μ*HM, exposing *μ*HM to a blocking dose of BaCl_2_ to inhibit *I_K_*_1_ led to a robust increase in beat rate regardless of tissue shaft length [Figs. 3(e) and S8(a)]. While the fold-increase in spontaneous beat-rate was statistically higher in 1 mm shaft-length *μ*HM compared to 0 mm *μ*HM, the effect size was modest, and a fold-increase in BaCl_2_-induced beat rate was not observed when compared 2 mm shaft length to 0 mm *μ*HM [Fig. 3(e)]. Moreover, 0 mm tissues showed a larger change in their APD_90_ compared to the two elongated tissues when exposed to BaCl_2_. These observations suggest that even if *I_K_*_1_ is regulated by cellular alignment and/or prestress, the effect size is too small for changes in Kir2.1 levels and/or trafficking to underlie the large changes we observed in STX-sensitive sodium currents [Figs. 3 and S8].

### Tissue geometry regulates sodium channel and gap junction protein expression

**FIG. 4.**
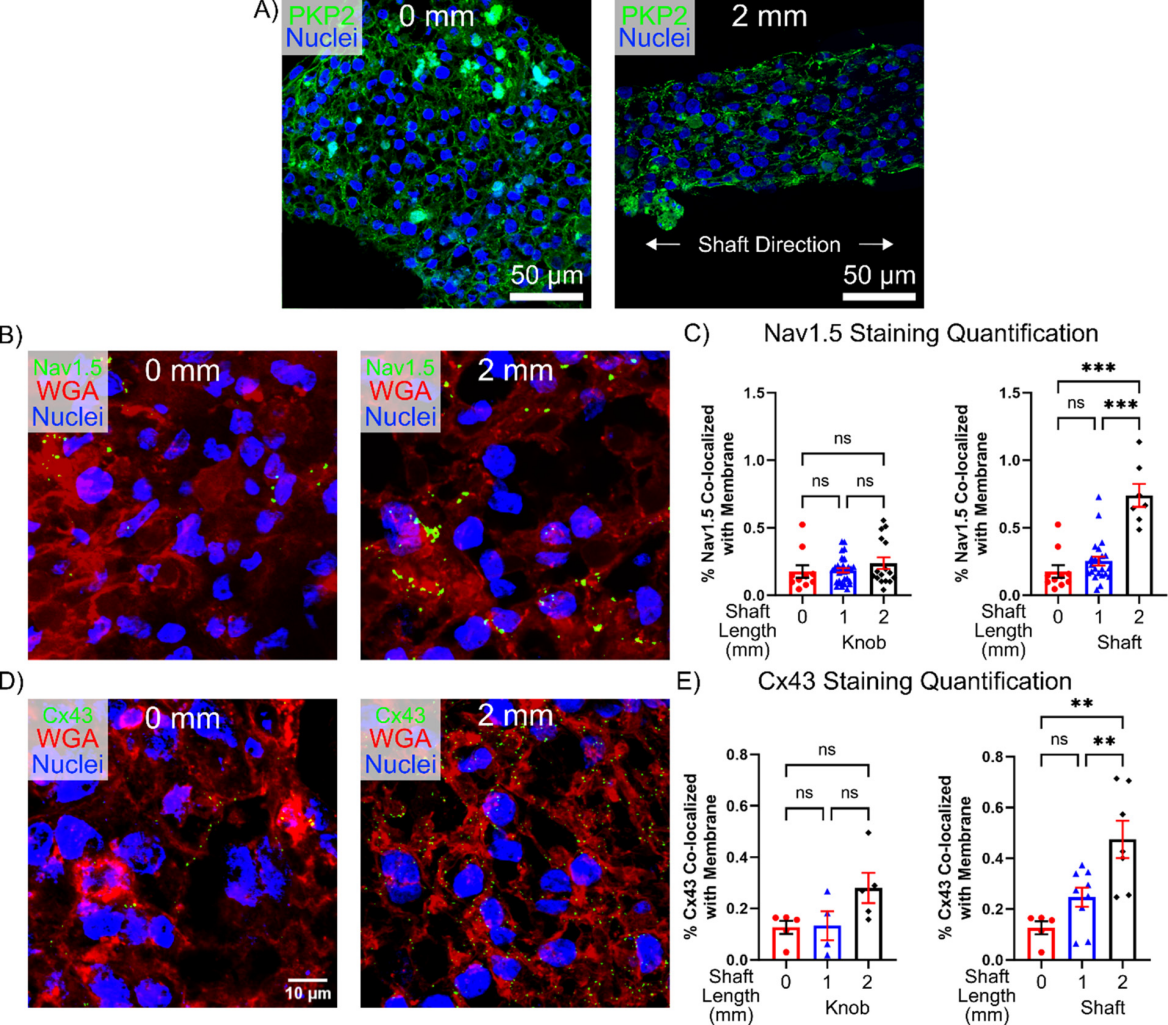
Immunostaining quantification of Nav1.5 and Cx43. Representative staining of 0 mm and elongated *μ*HM at *μ*D15 for: (a) Plakophilin-2, (b) Nav1.5, and (d) Connexin-43. Quantification of immunostaining for membrane localized protein: (c) Nav1.5 and (e) Cx43 in 0-, 1-, and 2-mm tissues at *μ*D15, separated to compare 0 mm tissue to the knobs or shafts of 1- and 2-mm tissues (*n* = 4–32 *μ*HM per size).

To identify potential molecular mechanisms causing differential *I_Na_* in *μ*HM, we assessed the levels and localization of plakophilin-2, Nav1.5 (sodium channel), and Connexin 43 (Cx43; a major gap junction in cardiomyocytes) proteins via confocal microscopic analysis of longitudinal *μ*HM cryosections cut near the vertical center of each tissue (Fig. 4). Plakophilin-2, a major component of the desmosome and associated with the function of both Na_V_1.5 and Cx43,^52^ was localized correctly to cell-cell junctions regardless of *μ*HM geometry [Fig. 4(a)]. Cx43 was chosen as it may play a role in the differences seen in the action potential upstroke duration [Fig. 2(c)], and prior work indicating that Cx43 regulates trafficking of sodium channels.^53,54^ Strikingly, total levels of both proteins increased proportionally within cardiomyocytes in the shaft region of *μ*HM as shaft length increased [Figs. S10(b) and S10(c)]. As with changes in cellular morphology, observations of increased Na_V_1.5 and Cx43 expression were limited to the shaft region of the *μ*HM [Figs. 4(c) and 4(e)]. Cells within knob regions of the same tissues exhibited only a weak trend toward higher expression of these proteins as shaft length increased [Figs. 4(c) and 4(e)]. In contrast to Na_V_1.5 and Cx43, Ki67, a marker for cell cycle entry, did not exhibit trends toward changes within different regions of elongated *μ*HM, or as a function of *μ*HM geometry (Fig. S12). As the cell cycle exit occurs during maturation of stem cell derived cardiomyocytes,^55^ this result suggests that regulation of sodium channel and gap junction levels and function can be triggered by cardiomyocyte alignment and/or prestress in the absence of global shifts of maturation state.

Consistent with our observation that the majority of change in Cx43 and Na_V_1.5 protein expression levels occurred in cells within the shaft region of *μ*HM, which occupies a substantially lower volume fraction of the tissues compared to knob regions [Fig. 1(b)], we observed no significant changes in a series of transcripts related to ion channel function and cardiomyocyte structure in whole-tissue lysates [Fig. S10(a)]. Likewise, Western analysis of bulk tissue lysates for both Nav1.5 and Cx43 protein levels did not show any trend toward upregulation as shaft length increased (Fig. S11).

### Generation of plakophilin 2 knockout micro-heart muscle arrays

**FIG. 5.**
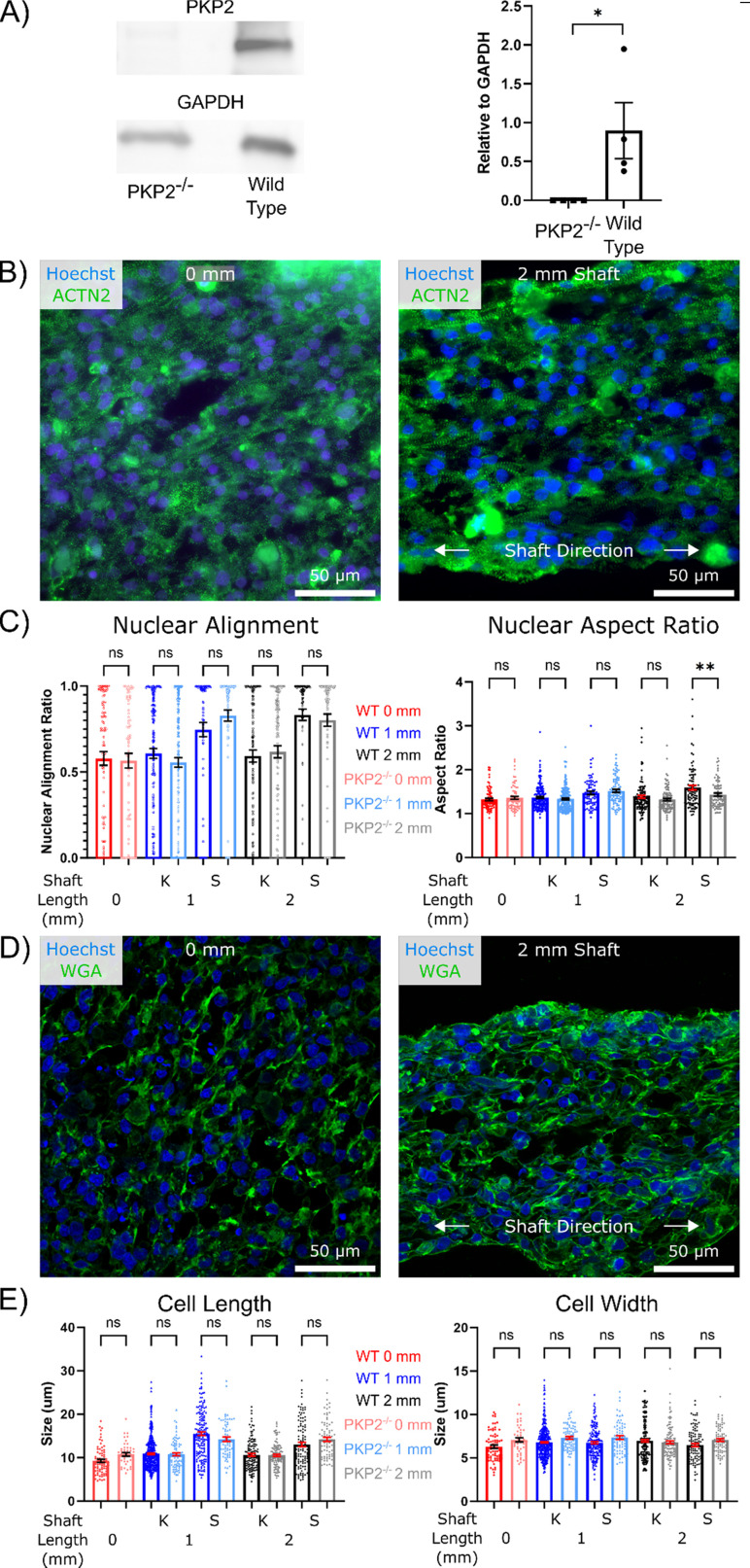
Loss of PKP2 does not inhibit prestress regulation of cell morphology (a) Representative western blot for plakophilin-2 in wild type and PKP2^−/−^ iPSC, and the resulting quantification from four blots. (b) Representative images of nuclei (Hoechst/blue) and sarcomeric α–actinin (green) of 0 and 2 mm PKP2^−/−^ *μ*HM at *μ*D15. (c) Quantification of the effects of tissue geometry on nuclear alignment and nuclear aspect ratio at *μ*D15 on PKP2^−/−^ *μ*HM in comparison to wild type tissues. (n > 70 nuclei from 6 to 8 *μ*HM per size). (d) Representative images of nuclei (Hoechst/blue) and cell membrane (wheat germ agglutinin/green) of 0 and 2 mm PKP2^−/−^ *μ*HM at *μ*D15. (e) Quantification of the effects of tissue geometry on cell length and width at *μ*D15 on PKP2^−/−^ *μ*HM in comparison to wild type tissues. (n >100 cells from 6 to 8 *μ*HM, ^*^: p < 0.05, ^**^: p < 0.005, ^***^: p < 0.0005, error bars: SEM).

Cardiomyocytes contain specialized mechanical junctions called desmosomes,^56^ which enable cell-cell connections within the high stress environment of the heart, and are comprised of: desmoglein-2, desmocollin-2, plakoglobin, plakophilin-2 (PKP2), and desmoplakin. Patients with genetic mutations in desmosome proteins, most commonly PKP2, suffer from arrhythmogenic cardiomyopathy (ACM), which is among the leading causes for sudden cardiac death in children.^37,38,57–60^ Prior studies point to dysfunction of sodium channels,^61^ gap junctions,^62^ and calcium handling^63^ in cellular and animal models of ACM. *In vitro* studies have demonstrated the ability to model genotype-phenotype relationships in PKP2 deficient iPS-CM, but these relationships required extensive efforts to mature cardiomyocytes through prolonged culture^64^ or other means including xenogeneic transplantation.^65^ Studies in engineered heart tissues have suggested depressed contractility of iPS-CM harboring desmosome protein mutations.^22,66^ However, defects in electrophysiological functions associated with ACM, like conduction velocity, were only realized using sophisticated devices that selectively applied diastolic tension (preload) to engineered tissues formed from iPS-CM with desmosome mutations.^67^ Based on our observations that variations in sodium channel function could be elicited over relatively short (<2 weeks) time for iPS-CM cultured in this *μ*HM format, we sought to determine if we could leverage these tissues to study genotype-phenotype relationships in PKP2 knockout iPS-CM. Thus, we used CRISPR/Cas9 to generate PKP2^−/−^ iPSC [Fig. S13(a)]. Absence of PKP2 protein in differentiated iPS-CM was confirmed via immunoblotting [Figs. 5(a), S13(b), and S13(c)].

### Loss of plakophilin-2 does not affect cell morphology within *μ*HM

Analysis of sarcomeric α-actinin, cell membrane, and nuclei in PKP2^−/−^ *μ*HM did not reveal any gross differences between PKP2^−/−^ iPS-CM and their isogenic control “wild type” (WT) counterparts in geometrically matched *μ*HM [Figs. 5(b)–5(e)]. Quantification of nuclear alignment suggested no significant differences in overall nuclear alignment between control and PKP2^−/−^ *μ*HM. A similar trend was observed with respect to nuclear aspect ratio, although the absolute level of nuclear anisotropy was slightly lower in 2 mm PKP2^−/−^ *μ*HM compared to 2 mm control tissues [Fig. 5(c)]. The correlation of increased tissue and cell length seen in WT tissues was also maintained in PKP2^−/−^ *μ*HM, along with the lack of change in cell width [Fig. 5(e)]. Consistent with continuum mechanics modeling predictions [Fig. 1(b)] and results from control tissues, changes to nuclear and cell morphology were most pronounced in the tissue shaft (Fig. S14). These observations are consistent with findings that PKP2 null mouse embryos are capable of forming the primitive heart tube^68^ and match previously published results where cardiomyocytes from adult mice in which PKP2 was conditionally knocked out maintain their size and overall shape.^63^ While likely incapable of withstanding the prestress levels present in postnatal or even late stage embryonic hearts, the PKP2 knockout iPS-CM generated here are still likely to retain other protein systems critical for cell-cell mechanical coupling, including cadherins and catenins.^69^ These alternative protein systems are likely sufficient to induce prestress-regulated morphology changes in these tissues despite the loss of PKP2, and the potential impacts of this loss on nascent desmosome structure and mechanical integrity.

### PKP2 knockout *μ*HMs exhibit exaggerated calcium intake in response to changes in tissue geometry

**FIG. 6.**
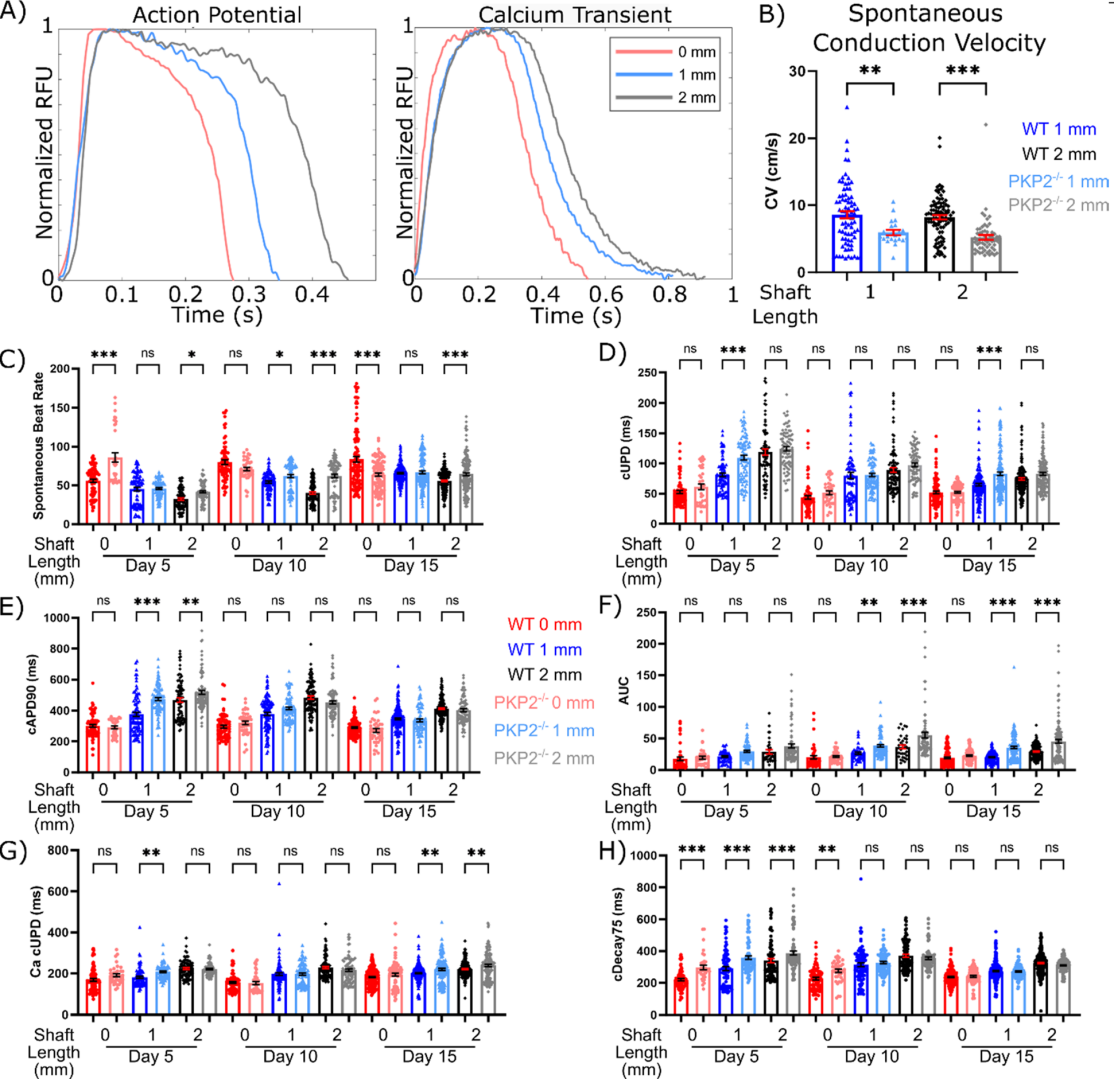
Loss of PKP2 causes minimal differences in gross electrophysiology. (a) Representative action potential and calcium transient waveforms for 0, 1, and 2 mm PKP2^−/−^ *μ*HM at *μ*D15. (b) Spontaneous conduction velocity for PKP2^−/−^ *μ*HM at *μ*D15, compared to WTC tissues. (c) Spontaneous beat rate, (d) beat-rate corrected action potential upstroke duration, (e) APD90, (f) calcium transient area under the curve, (g) calcium upstroke duration, and (h) Decay75 for 0, 1, and 2 mm PKP2^−/−^ *μ*HM, compared to WTC tissues (n = 40–100 *μ*HM, ^*^: p < 0.05, ^**^: p < 0.005, ^***^: p < 0.0005, error bars: SEM).

We next investigated the role that PKP2 plays in the prestress-regulated electrophysiology changes we observed in control *μ*HM. Like the isogenic control, PKP2^−/−^ *μ*HM exhibited global remodeling of action potentials and calcium transients as shaft length increased [Fig. 6(a)], Quantitative analysis of action potential kinetic parameters further suggested no significant differences between control and PKP2^−/−^ *μ*HM at day 15 [Figs. 6(c)–6(e) and S15]. Slight differences were observed in the spontaneous beat rate and action potential upstroke duration between tissues of the two genotypes [Figs. 6(c) and 6(d)]. However, these small differences were not observed consistently across all shaft lengths and time points.

Whereas they showed similar global action potential and calcium remodeling in response to changes in tissue geometry, PKP2^−/−^ *μ*HM exhibited substantially lower conduction speed than isogenic control *μ*HM [Fig. 6(b)]. Similar observations of impaired conduction have been reported in neonatal rodent cardiomyocytes and iPS-CM cultured in which desmosome constituents were knocked down with siRNA.^70,71^ Interestingly, despite having overall similar action potential waveform kinetics, PKP2^−/−^ *μ*HM did show an increase in the calcium transient upstroke duration, and thus the total calcium flux (AUC) as well, for both the 1- and 2-mm *μ*HM in comparison to control tissues with the same geometry [Figs. 6(f) and 6(g)]. The increase in the calcium influx via increased calcium transient upstroke time correlates with previous observations from cardiomyocytes derived from conditional PKP2 knockout adult mice, although we did not see an increase in Ca^2+^ transient decay time, which was reported in that system.^63^

### PKP2 deficient *μ*HMs lack functional sodium currents

**FIG. 7.**
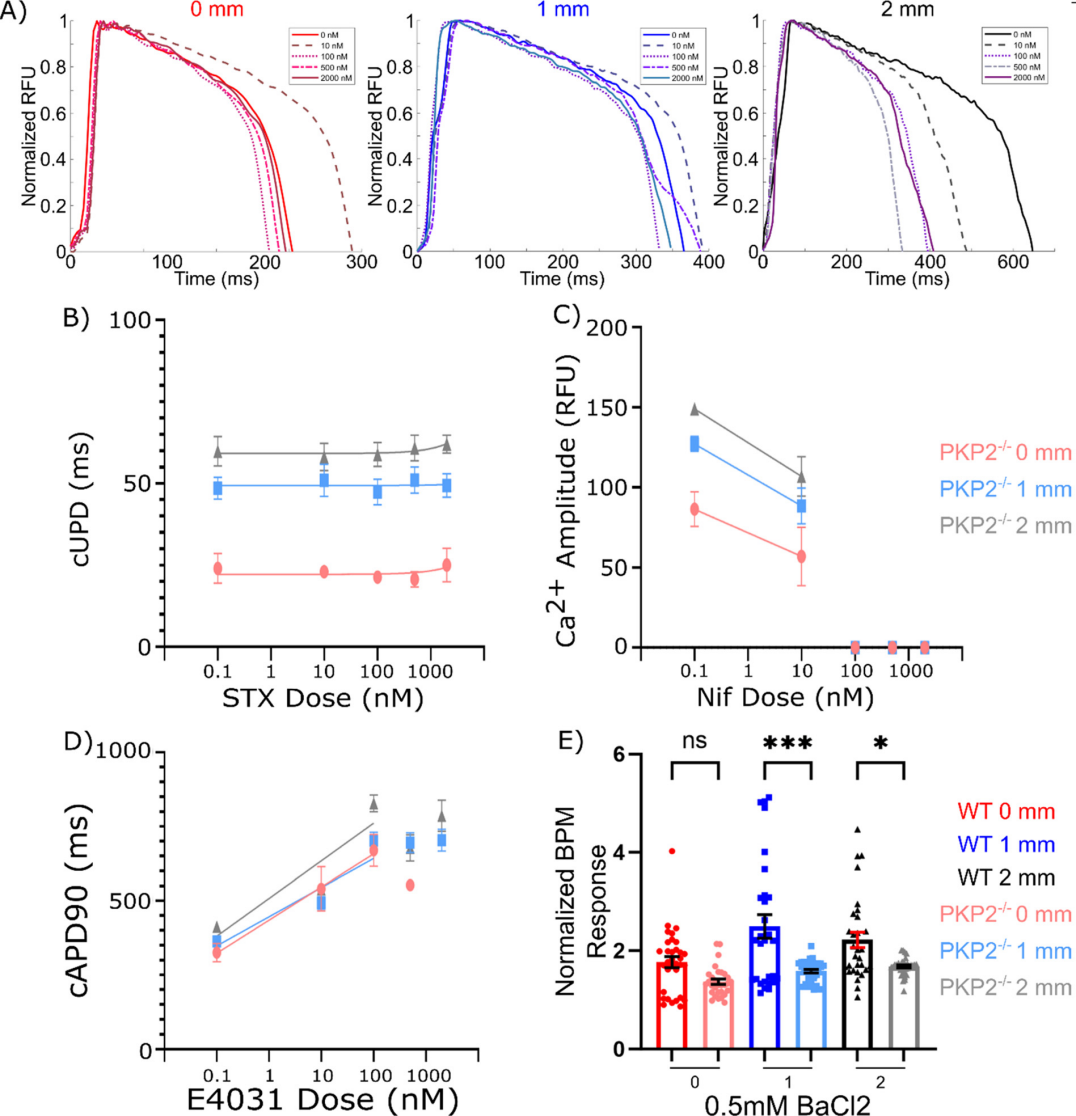
Pharmacologic inhibition of ion channels in PKP2^−/−^ *μ*HM. (a) Representative action potential waveforms for 0, 1, and 2 mm PKP2^−/−^ *μ*HM after sequential saxitoxin doses. Response of PKP2^−/−^ *μ*HM to pharmacologic inhibition of ion channels. Effect of (b) saxitoxin on action potential upstroke duration, (c) nifedipine on calcium transient amplitude, (d) E4031 on ${\mathrm{cAPD}}_{90}$, and (e) 0.5 mM BaCl_2_ on spontaneous beat rate. (*n* = 20–30 *μ*HM, ^*^: p < 0.05, ^**^: p < 0.005, error bars: *SEM*).

Unlike control *μ*HM, which exhibited marked action potential morphology changes when *I_Na_* was blocked with saxitoxin, PKP2^−/−^ *μ*HM exhibited no STX-linked changes to action potential upstroke duration or amplitude and exhibited minimal changes in the spontaneous beat rate, regardless of tissue geometry [Figs. 7(a), 7(b), and S16(a)].

Complete inhibition of Ca_v_1.2 at 100 nM nifedipine had a similar effect in PKP2^−/−^ *μ*HM as in isogenic controls, which led to a complete block of action potential initiation [Fig. 7(c)]. Strikingly, however, while 10 nM of nifedipine had minimal effects on the amplitude of the calcium transients for control *μ*HM [Fig. 3(c)], this dose markedly decreased Ca^2+^ transient amplitude in PKP2^−/−^ tissues. The slope of this decrease appears relatively similar for all PKP2^−/−^ tissue sizes, suggesting similar Ca_v_1.2 kinetics. Likewise, while 10 nM of nifedipine led to no difference in the upstroke duration of the calcium transient in control *μ*HM [Fig. S6(c)], this led to a significant decrease in PKP2^−/−^ tissues [Fig. S16(c)]. These results suggest the higher action potential cUPD for the 2 mm PKP2^−/−^ tissues is due to increased calcium influx through L-type channels, as *I_Na_* makes no contribution to the action potential upstroke in these PKP2 deficient tissues.

**FIG. 8.**
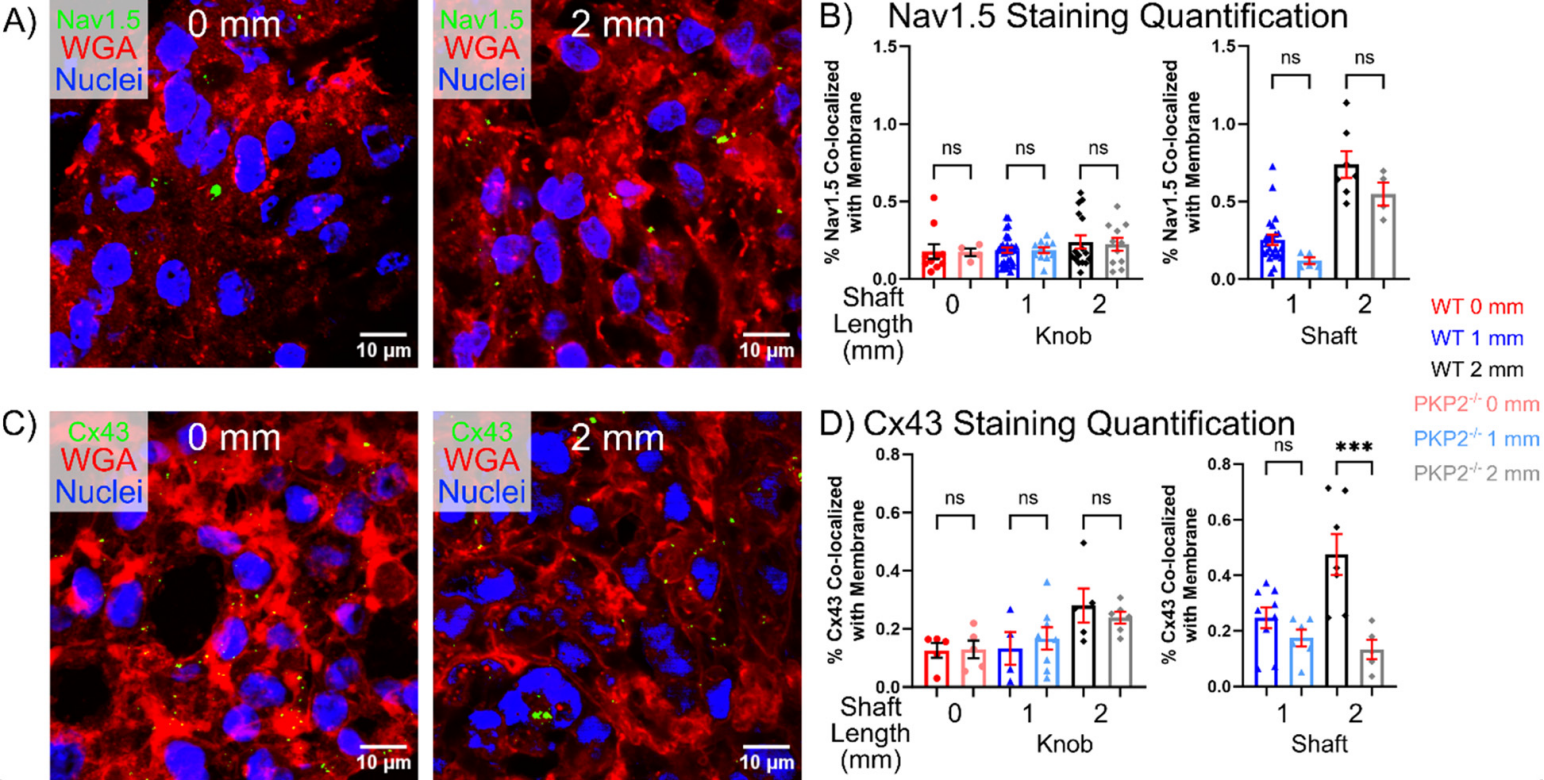
Immunostaining quantification of Nav1.5 and Cx43 in PKP2^−/−^ *μ*HM. Representative staining of (a) Nav1.5 and (c) Cx43 in 0 and 2 mm PKP2^−/−^ *μ*HM at *μ*D15. Quantification of immunostaining for total membrane localized protein: (b) Nav1.5 and (d) Cx43 in 0, 1, and 2 mm PKP2^−/−^ *μ*HM compared to wild type tissues at *μ*D15, separated by 0 mm compared to the knobs of the elongated tissue, and the shaft regions of the elongated tissues. (*n* 4–32 *μ*HM per size).

Blockage of hERG with E4031 showed a nearly identical effect on the cAPD_90_ of PKP2^−/−^ *μ*HM as in WT *μ*HM [Fig. 3(d)], with a dose of 500 nM and above inhibiting the ability for cardiomyocytes to repolarize [Fig. 8(d)]. Additionally, the slope of the sensitivity across all tissues appears the same, suggesting minimal differences in hERG activity across *μ*HM shaft lengths. However, a dose of 100 nM significantly decreased the spontaneous beat rate in PKP2^−/−^ *μ*HM [Fig. S18(a)], whereas the effect of this dose on WT *μ*HM was minimal [Fig. S7(a)]. A similar effect was seen on the action potential amplitude as well.

Finally, we examined potential changes in *I_K_*_1_ in PKP2 deficient *μ*HM via a blocking dose of BaCl_2._ Although PKP2^−/−^*μ*HM of all shaft lengths exhibited some changes in their spontaneous beat rate in the presence of *I_K_*_1_ block, the degree of these changes was less than those observed in control *μ*HM [Fig. 7(e)]. Changes to other waveform parameters such as action potential duration and calcium transient decay appeared similar to changes in isogenic controls (Fig. S19).

Altogether, these observations suggest that PKP2 deficient cardiomyocytes retain an ability to adapt to changes in prestress and/or cell alignment-based regulation by regulating action potential waveforms. However, electrophysiologic adaptation appears to be driven primarily by changes in activity of *L*-type calcium channels rather than sodium currents.

### PKP2 deficient cardiomyocytes exhibit deficits in gap junction protein expression

Given their depressed conduction velocity and lack of STX-responsiveness, we hypothesized that PKP2^−/−^ *μ*HM may lack expression of Na_V_1.5 and/or Cx43, which others have reported to be deficient in experimental models of ACM.^72^ While we observed a trend toward decreased Na_V_1.5 expression in PKP2^−/−^ cardiomyocytes within the shaft region of *μ*HM [Fig. 8(b)], this trend was not statistically significant between genotypes (*p* > 0.07 comparing between genotypes in 1 mm *μ*HM and *p* > 0.16 comparing between genotypes in 2 mm *μ*HM, 2-way *t*-test) and was not strong enough to explain the significant reductions in conduction speed and the lack of STX response. However, Cx43 protein levels within PKP2^−/−^ cardiomyocytes were significantly lower than in isogenic control counterparts within the shaft region of 2 mm *μ*HM [Fig. 8(d)], correlating with results seen in ACM patients.^73^ Bulk quantification of Na_V_1.5 protein levels by western blot over entire *μ*HM lysates suggested no trends toward differences when comparing the 2 mm PKP2^−/−^ tissues to the wild type tissues of any size [Fig. S20(a)]. As with observations in control *μ*HM, this is likely because most of the volume of *μ*HM is contained within the knobs, which did not show any trend toward differences in expression of as a function of genotype (Fig. S11). Quantification of Cx43 protein levels in PKP2^−/−^ tissues also showed no differences across the different tissue sizes [Fig. S20(b)], like the results seen for WT tissues (Fig. S11). Altogether, these results suggest that PKP2 null and isogenic control iPS-CM have similar baseline expression levels of Na_V_1.5 and Cx43 and further that PKP2 deficient iPS-CMs are capable of adapting morphologically to different tissue geometries and concurrent changes in cellular alignment mechanical prestress. However, as was previously observed by Ng *et al.*,^67^ desmosome proteins like PKP2 appear to be required for proper electrophysiologic adaptation to biophysical stresses like prestress and preload.

## CONCLUSIONS

Human induced pluripotent stem cell-derived cardiomyocytes show promise in predicting drug-induced arrhythmias with more accuracy compared to alternative *in vitro* assays.^1^ Tissue engineering approaches have been applied in an effort to enhance iPS-CM electrophysiologic maturity.^2,14,16,20,21,31,74^ To test the hypothesis that prestress on and/or cellular alignment of cardiomyocytes in the 3D setting of engineered tissues plays a role in electrophysiologic development, we tested the impact of micro-tissue geometry on action potential and calcium transients. Our observations reveal cellular prestress and/or alignment regulate activity of specific ion channels to drive remodeling of the cardiac action potential. Importantly, these mechanical effects are sufficient to drive a lower spontaneous beat-rate and form functional Na_V_1.5 current after only two weeks of culture. It is likely that these changes are due to increased levels of tissue prestress and not cellular alignment, as previously published studies comparing monolayer vs linear patterned iPS-CM on 2D substrates did not lead to the changes in APD and calcium transient upstroke and decay times that we observed when comparing 0, 1, and 2 mm *μ*HM.^75,76^ This may be due to the tendency of strong cell-ECM adhesions to diminish the tension transmitted across cell–cell junctions when cultured in 2D formats,^77^ helping explain why 3D tissues are critical for improving stress-regulated iPS-CM maturity. However, no level of prestress and/or cellular alignment was able to eliminate spontaneous beating at this timepoint, and action potential upstroke is still Ca^2+^ dependent. These observations suggest that applying additional exogenous stretch and/or combining geometric constraints with chemical and chemical stimuli may be required to induce further electrophysiological maturation.^26,78^

3D culture of iPS-CM within *μ*HM with elongated shafts, particularly the longer 2 mm shaft, elicited robust sodium function along with expression of Na_V_1.5 and Cx43 over relatively short culture timeframes. This prompted us to leverage this system to study the impact of PKP2 deficiency, which is linked to inherited cardiomyopathy.^36^ PKP2 null *μ*HM exhibited several hallmarks that have been reported by other groups studying PKP2 deficiency in cultured cardiomyocytes and *ex vivo* adult rodent cardiomyocytes, including depressed conduction, inhibited sodium channel function,^70^ and exaggerated calcium intake.^63^ iPS-CMs within PKP2^−/−^ *μ*HM were morphologically similar to iPS-CM in WT control tissues, correlating with previously published results using an alternative engineered heart tissue design in which desmoplakin deficient tissues did not show any differences in their diastolic length compared to WT tissues.^22^

The *μ*HMs in our study are formed on rigid tissue culture plastic, and thus contract in an isometric manner, precluding analysis of tissue or cellular shortening. Studies by other teams suggest a general trend toward hypocontractility in iPS-CM based engineered heart tissues with loss-of-function mutations in PKP2 and desmoplakin,^22,66^ whereas Ng *et al.* reported hypercontractility with a desmoplakin mutation.^67^ In future studies, it would be interesting to combine PKP2^−/−^ *μ*HM with compliant substrates^23^ to examine contractility and excitation-contraction coupling.

Exaggerated calcium intake of adult mouse cardiomyocytes harboring conditional PKP2 deletion was concurrent with calcium sparks,^79^ which are strongly linked to cardiac arrhythmia.^80^ The high cell density of *μ*HM precludes us from assessing similar events within intact tissue. Nevertheless, it is noteworthy that iPSC-cardiomyocytes at a relatively primitive stage of development could elicit similar PKP2-genotype linked electrophysiologic behaviors as adult rodent cardiomyocytes if the iPS-CM were subjected to appropriate culture conditions. Potentially, changes in calcium channel levels and/or activity that led to increase Ca^2+^ intake in our study [Figs. 6(f) and 6(g)] and in prior work by others^63^ allow PKP2 deficient cardiomyocytes to compensate for their lack of sodium current, as these cells adapt to increases in mechanical stress conferred by culture within elongated tissues *in vitro* and within hearts subjected to normal physiologic stresses *in vivo*.

In the present study, tissue geometry and genomic dose of PKP2 appeared to regulate action potential waveforms via changes in expression of sodium channel (Na_V_1.5) and gap junction (Cx43) proteins. However, changes in the levels of these proteins could only be observed within regions of the *μ*HM where cellular morphology was remodeled. Changes in expression and localization for both Na_V_1.5 and Cx43 have been previously linked to genomic variants of PKP2 or other desmosome proteins,^63,67,70,71^ as well as to other cellular structures (e.g., microtubules and focal adhesions) commonly associated with mechanotransduction.^81–85^

We observed strong coupling between the total levels and membrane localized levels of both these proteins within our studies for both WT and PKP2^−/−^ *μ*HM (Figs. 4, S9, and S10), suggesting against gross changes in trafficking of either protein as a potential mechanism for our observed changes in physiology. It is possible, however, that higher precision measurements for localization, as provided by super-resolution microscopic approaches,^84^ might reveal more subtle changes in trafficking of Na_V_1.5 and/or Cx43. Proper trafficking of Cx43 is dependent on the GJA1-20k isoform.^86^ A similar mechanism exists for Nav1.5, in which the β2 subunit regulates trafficking to the cell membrane.^87^ Cellular localization of both Na_V_1.5 and Cx43 may play a role in degradation/recycling and, thus, total steady-state levels measured for both proteins.^88^

Prior work by Sengupta *et al.* suggests that changes in focal adhesion assembly can drive changes in localization of Kir2.1,^82^ which in turn might regulate the function of sodium channels.^50,51^ However, in contrast to our observations for changes in STX-sensitive *I_Na_*, we observed very little change in BaCl_2_-sensitive *I_K_*_1_ as a function of *μ*HM geometry. This suggests the possibility that cellular alignment and/or prestress may regulate Na_V_1.5 expression through at least a subset of mechanisms that are independent of Kir2.1. Recently published by other groups suggest the cytoskeletal microtubule network plays crucial roles in enabling proper trafficking of ion channels such as Nav1.5,^89,90^ which may partially explain the mechanism by which tissue prestress and plakophilin-2 regulate Nav1.5 functionality. Others have implicated a direct role for desmosomes in signaling that regulates cellular degradation of Cx43, providing another means for such regulation.^91^

Ultimately, our findings suggest that a threshold level of cellular prestress and/or alignment, which can be controlled simply by manipulating the geometric boundary conditions under which engineered heart tissues form, is required for expression of Na_V_1.5 and subsequent sodium channel function. This is sufficient to model the impact of PKP2 deficiency on tissue conduction over short culture timeframes in simple, scalable engineered tissue formats. This work suggests that cardiomyocytes adapt to increased levels of tissue prestress, a surrogate for *in vivo* preload, by increasing their calcium influx and handling, which is achieved by increasing the sodium current to increase the ion influx into the cell. By extension, engineered heart muscle designs that subject cells within to higher levels of prestress during tissue compaction may yield differences in electrophysiologic remodeling, although it is still uncertain whether these can achieve remodeling to the extent that is observed when tissues are subjected to exogenous stretch.^67,78^

A limitation of the *μ*HM system is that the bulk of the volume of the tissue is within regions predicted to be subjected to relatively low prestress during tissue compaction [Fig. 1(b)]. As these “stress shielded” knob regions of the tissue showed significantly less cell morphology and protein expression changes, this design limitation precluded detailed analysis of bulk-tissue changes in proteins and transcripts that might further clarify mechanisms for sensing cellular alignment and/or prestress. Engineered tissue designs in which a larger volume fraction of the tissue experiences mechanical stress during tissue compaction may be better suited for such analyses in future studies. Additionally, although we observed increased calcium influx with increasing shaft length in wild-type *μ*HM [Fig. 2(e)], as well as increased influx when comparing PKP2^−/−^ *μ*HM to isogenic control tissues of the same size [Figs. 6(f) and 6(g)], our current system did not allow us to study any potential changes to the active contraction of these tissues. It would be of interest to measure the effects of tissue geometry and prestress on contractile tension given the correlation between calcium influx and contractile strength^48^ and the associated improvement in cardiomyocyte maturity.^46,47^

Beyond the extension of the study of tissue geometry to cardiomyocyte electrical development in other tissue geometries, future extension of this study can focus on how dynamic changes in tissue stretch cardiomyocyte physiology.^13,67,78^ Incorporating this with systems that apply differential levels of afterload^23^ to determine how they independently regulate electrophsyiology would also be of interest. Previously published studies utilizing other engineered heart tissue systems that incorporate electrical pacing have described the formation of t-tubules and higher levels of tissue conduction velocity.^20,21^ Other work has utilized tissue designs that more accurately mimic the type of loading cardiomyocytes are subjected to *in vivo*, in which an afterload threshold must be overcome before tissue shortening can occur.^22^ It is unknown how these different types of loading regulate specific ion channel expression, or how the changes they induce in cardiomyocytes differ from the results seen here. It would be of great benefit to the field to understand how these different types of loading regulate physiology in unique ways.

## METHODS

### Stem cell-derived cardiomyocyte production

Wild Type C″ human induced pluripotent stem cells (iPSCs) were created at the Gladstone Institutes of Cardiovascular Disease (Coriell Institute GM25256) and were modified via knock-in of a single copy of GCaMP6f into the AAVS1 “safe harbor locus.”^26^ iPSCs were cultured at 37 °C in Essential 8 media on six-well plates coated with growth factor-reduced Matrigel (Corning). Once 85% confluency was reached, the cells were passaged into new wells using Accutase. iPSC were differentiated into cardiomyocytes using small molecule manipulation of Wnt signaling.^40^

The PKP2^−/−^ iPSCs were generated from wild-type C (WTC) iPSC by the Genome Engineering & Stem Cell Center (GESC@MGI) at Washington University in St. Louis School of Medicine. Briefly, gRNAs were designed to target exon3, an early exon where out-of-frame indels result in premature stop codons, triggering nonsense-mediated decay of PKP2 mRNA. Two gRNAs with target sequences, 5′-CATATCTCGGTGGCACTAGGAGG and 5′-TGCTCGTTCCGAGATCGTGGTGG were purchased as synthetic crRNAs from Integrated DNA Technologies (Coraville, IA), annealed to tracrRNA (also from IDT) and complexed with recombinant Cas9 protein. The formed ribonucleoprotein complexes (RNPs) were validated for cleavage activity in K562 cells, and the gRNA with higher activity was used to edit the parental line: 5′-CATATCTCGGTGGCACTAGGAGG [sp110 in Fig. S13(a)]. The WTC AAVS1-CAG-GCaMP iPS cells were nucleofected with RNP, and single cell iPSC clones were screened for biallelic knockout of the PKP2 gene using amplicon NGS. The clone chosen has 14 and 23 bp deletions in respective alleles. The knockout was confirmed by next-gen sequencing (data not shown) and western blot [Figs. 6(a) and S13(b)].

### *μ*HM-Forming Stencil Fabrication

Using Solidworks, the stencil mold was created by first extruding a rectangular base several millimeters thick to prevent warping during the polymer cross-linking process, and eventual print removal from the printer. On top of this base an individual dogbone shape is designed and extruded to a height of 1 mm. Individual dogbone-shaped molds are clustered in groups of 3 with several millimeters of spacing between groups. The individual dogbone shapes have a 1 × 1 mm^2^ knob/square on each end, with a 400 *μ*m wide shaft of variable length.

The mold was replicated into PDMS using Hydrogel Assisted STereolithographic Elastomer prototyping (HASTE) as previously described.^39^ Briefly, a negative of the 3D-printed mold is first created by casting off agar. Sylgard 184 (Dow Corning) is mixed according to the manufacturer's instructions, poured onto the agar negative, degassed, and cured at 37 °C for ≥8 h to form a PDMS positive of the 3D-printed mold.

The PDMS replicates of the 3D-printed molds were oxidized using air plasma (Harrick Plasma) for 90 s at high power. These were then treated with trichloro(1H,1H,2H,2H-perfluorooctyl)silane via vapor deposition to enable PDMS-off-PDMS molding for final stencil creation. Stencil molds with “dogbone” shaped through-holes were formed by pouring Sylgard 184 over the treated PDMS molds, clamped between glass/acrylic plates and cured at 60 °C for 4+ h. Each dogbone shaped through-hole is 1 mm thick, and three different dogbone sizes were created: (1) a tissue that is just a 1 × 1 mm^2^ knob with no shaft (“0 mm”), (2) a tissue with 1 × 1 mm^2^ knobs and a 400 *μ*m × 1 mm shaft (“1 mm”), and (3) 1 × 1 mm^2^ knobs and a 400 *μ*m × 2 mm shaft (“2 mm”) [Figs. 1(b) and 1(c)].

### Finite Element Modeling

To predict prestress developed within tissues as they compact, we performed finite element modeling using COMSOL Multiphysics 6.1. In this model [Fig. S1(a)], the dimension of initial boundary condition is created as in the cell-based experiments, where molds with varying dimensions are filled with cells. The initial height (z-direction) was set to 500 *μ*m. The tissue shape was set either to a 1 × 1 mm^2^ knob only (0 mm shaft), or two 1 × 1 mm^2^ knobs connected by a 400 *μ*m wide shaft with a length of either 1 or 2 mm. The mass of cells was modeled as a Neo-Hookean material, with 10 kPa Young's Modulus, 970 kg/m^3^ density and a Poisson's ratio of 0.49. Based on prior observations that tissue within knobs tends to remain firmly attached to the substrate, whereas free tissue compaction is observed in the shaft region,^14,23^ the lower boundary of each knob was fixed, and the rest of the tissue was allowed to undergo a 50% volumetric compaction (isotropic shrinkage).

The mechanical consequences of shrinkage against these boundary conditions were modeled with a mesh consisting of 414 092 tetrahedral domain elements, 24 138 boundary elements, and 1248 edge elements, ranging from 0.018 to 0.05 *μ*m size. After obtaining simulated stress, we extracted the surface Solid von Mises stress throughout the tissues. The heatmap in Fig. S1(a) shows the normalized to the highest value of surface stress from the 2 mm shaft. For quantitative data, we provided the line graphs showing the stress at 0.1 mm z-position from the base, at the center of the x–y plane [Figs. S1(b)–S1(d)].

This modeling predicted stepwise increases in tissue prestress within the center of the tissue shaft. These parameters were chosen based on prior experimental observations on *μ*HM formation and compaction after initial cell seeding. During tissue seeding the single-cell cardiomyocyte/media suspension fills the entirety of the dogbone-shaped stencil. After 24 h, the cells have self-compacted and assembled to form the *μ*HM. After tissue compaction, the tissue remains adherent to the substrate, in this case tissue culture plastic, while the shaft region is lifted and non-adherent to the substrate nor the PDMS stencil. Based on observations of the *μ*HM before and after tissue formation, it appears the cells compact isotropically, consistent with the input provided to the COMSOL model.

### *μ*HM Formation

PDMS stencils were cut, dipped in methanol, and placed into the wells of tissue culture well plates. The well plate was then placed into a 60 °C oven overnight to reversibly bond the PDMS stencil to the well surface.^92^ The well was then coated with fibronectin to enable cardiomyocyte attachment to the substrate.

iPS-CM at day 15 of differentiation were singularized using 0.25% trypsin and seeded at a density of 7.5 × 10^7^ cells/ml at 3 *μ*l per individual *μ*HM (∼225,000 cells per tissue) without exogenous extracellular matrix. Seeded cells were incubated at 37 °C for 30–60 min before adding media to limit cell loss, using DMEM with 20% FBS, 10 *μ*M Y-27632, 150 *μ*g/ml L-ascorbic acid, 4 *μ*g/ml Vitamin B12, and 3.2 *μ*g/ml penicillin. *μ*HM typically began spontaneous beating within 24–48 h of seeding; upon observing beating tissues the media was changed to RPMI/B-27 supplemented with 150 *μ*g/ml L-ascorbic acid, 4 *μ*g/ml Vitamin B12, and 3.2 *μ*g/ml penicillin (collectively called R+ media). *μ*HM were then fed R+ media every 2–3 days until termination.

### Immunohistochemistry

Tissues were fixed using increasing concentrations of paraformaldehyde from 1% to 4%.^20,23^ PDMS stencils were then removed, and tissues were embedded in 1% agar. Agar-embedded tissues were then cryoprotected using 15% and 30% sucrose, flash frozen in OCT, and cryosectioned at 8–15 um. Sectioning was done parallel to the tissue longitudinal axis. Tissue sections with an approximately “full-width” tissue section were chosen for imaging. These sections were in the middle 50% of the z-axis of the tissues, although the exact z-position of these sections within the tissues was not recorded. Samples were permeabilized with 0.1% Triton-X-100 for 20 min and blocked using 5% BSA in 0.1% Triton-X-100 for 45 min at room temperature. Primary antibodies were incubated overnight at 4 °C, secondary antibodies were incubated at room temperature for 2 h, and cell nuclei (ThermoFisher Hoechst 33342) were stained at room temperature for 10 min. The samples were then mounted with ProLong Gold (Invitrogen P36930) and imaged on an Olympus confocal microscope (Fluoview FV1200, Tokyo, Japan) using a 60X 1.35NA objective in XY scan mode at a sample speed of 20.0 *μ*s/pixel. Confocal scans were performed using line sequential mode, with line Kalman integration on with a count of 10–12. Specific primary antibodies are listed in Table S1.

### Cell morphology Quantification

All images were quantified using ImageJ. Nuclear aspect ratio was quantified as the nuclear major axis length divided by the minor axis length, where a value of 1 indicates a perfectly spherical nucleus. Nuclear alignment was quantified as the percentage of nuclei whose major axis aligned along the same vector, where a value of 1 indicates the major axis of all nuclei were perfectly parallel to each other. Nuclei with an aspect ratio > 0.9 were assigned an alignment score of 0 as an alignment for a sphere could not be quantified. Cell length and width were quantified by staining the cell membranes with wheat germ agglutinin (ThermoFisher). Cell size was manually quantified in ImageJ by drawing straight lines to measure the cell length and width (Fig. S2).

### Ion Channel Localization Quantification

The images were acquired using 6 Z slices with a 12 step Kalman filter. A mask was used created from the WGA staining. For Cx43/Nav1.5 quantification of membrane localized staining, only protein that overlapped with this mask were used. The resulting protein was then converted to binary values, and the total area of the particles in the remaining image were quantified. A schematic of the protocol is shown in Fig. S9.

### Quantitative PCR

Total RNA was purified from tissues according to the manufacturer's instructions using RNAqueous total RNA isolation kit (Invitrogen AM1912). Additional steps were conducted to inactivate DNases. cDNA was then synthesized from the resulting RNA using oligo(dT)20 and Super-Script III (Invitrogen 18080051).

Genes were quantified by real-time PCR using SYBR Green primers (Table S2; MilliporeSigma) and was conducted on Applied Biosystems Step One Plus. Data analysis was carried out using log twofold change normalized to glyceraldehyde-3-phosphate dehydrogenase (GAPDH) gene expression.

### Western Blot

Tissues were lysed in commercial RIPA buffer (Alfa Aesar J63306), with protease inhibitor (Millipore-Sigma P8340) and Triton-X-100 (Fisher BioReagents BP151) added. Samples were incubated with a reducing agent (Invitrogen NP0009) and buffer (Invitrogen B0007) at room temperature for 20 min and loaded into 4%–20% polyacrylamide gels (Bio-Rad #4561094) and transferred onto a PVDF membrane (Immobilon-P IPVH00005).

Blots stained for Nav1.5 were blocked for 5 min at room temperature in EveryBlot Blocking Buffer (Biorad #12010020). Immunoblotting was performed overnight at 4 °C using the following primary antibodies: Rabbit anti-NaV1.5 at a 1:1000 dilution (Cell Signaling Technology #14421) or rabbit anti-GAPDH at a 1:2000 dilution (Cell Signaling technology #2118). Secondary detection was performed for 1 h at room temperature using goat anti-rabbit horseradish peroxidase-conjugated antibody at a 1:5000 dilution (Abcam ab6721) and Clarity Western ECL Substrate (BioRad #1705061). All wash steps were performed with TBS with 0.05% tween (TBS-T) at room temperature. Imaging was performed using a Biorad ChemiDoc MP Imaging System.

Membranes stained for PKP2 were blocked overnight at 4 °C with 5% milk powder in tris-buffered saline (TBS) and then stained overnight at 4 °C using the PKP2 primary antibody at a 1:100 dilution in 5% milk powder in TBS with 0.2% Tween-20 (TBS-T). Secondary antibody staining for these two proteins occurred at room temperature for 1 h in 5% milk powder/TBS-T. All washing steps occurred at room temperature in 5% milk powder/TBS-T. Membranes stained for Cx43 and GAPDH were blocked for 1 h at room temperature in 5% bovine serum albumin in TBS. Primary and secondary antibody staining for these two proteins occurred for 1 h at room temperature, with the antibodies diluted in TBS-T. The Super-Signal West Dura substrate was used for detection (Thermo Scientific 34076) on a Syngene PXi. All densitometry analysis was performed using ImageJ and normalized to GAPDH density.

### Optocardiography

These cells were previously genetically modified to express GCaMP6f to image calcium handling dynamics,^93^ and Berst-1 voltage sensitive dye^94^ was used to visualize the action potential. Tissues were imaged on a Nikon Eclipse Tsr2 inverted microscope equipped with a Hamamatsu ORCA Flash 4.0 V3 digital CMOS camera and a Lumencor AURA light engine. A Tokai thermal plate was used to maintain *μ*HM temperature during imaging. Videos were imaged at 200–450 fps. The MATLAB Bio-Formats package was used along with custom MATLAB software^41^ to calculate waveform parameters. To calculate conduction velocity, a modified version of the open-source MATLAB software Rhythm 1.0 developed by Laugher *et al.* was used.^95^

### Pharmacology Study

Saxitoxin (SRM NIST, Product # 8642a) was received in a solution of 80% acidified water (pH 3.5)/20% ethanol. Nifedipine (Sigma-Aldrich, Product #N7634) was initially dissolved in dimethyl sulfoxide. E4031 (Alomone Lab, Cat. #E-500) was initially dissolved in MilliQ water. The maximum percentage of DMSO in the total media for tissues treated with nifedipine was 0.02% at 2 *μ*M of drug, which was considered negligible. BaCl_2_ (Sigma 202738) was initially dissolved in MilliQ water to 100 mM.

All drugs were dissolved into phenol red free RPMI1640/B27 at 10 times the desired final concentration, and added to tissues by changing 10% of the media to limit potential thermal and mechanical agitation from full media changes. Tissues treated with saxitoxin, nifedipine, or E4031 were allowed to equilibrate for 15 min at 37 °C before imaging and were tested at the following final concentrations: 10 nM, 100 nM, 500 nM, and 2 *μ*M. Tissues treated with BaCl_2_ were allowed to equilibrate for 35 min at 37 °C before imaging and were tested at a final concentration of 0.5 mM BaCl_2_.

### *μ*HM Field Pacing

Fridericia's formula [Eq. (1), which depicts beat-rate correction for APD_90_] was used to beat-rate correct waveform parameters to 60 bpm^42^

cAPD90=APD90RR13, (1)

where RR is the RR-interval (60/beat rate in beats per minute). In our prior study, we observed no substantial difference in beat rate correction by Fridericia's formula vs other formulas used for iPS-CM and clinical ECG.^41^

To determine whether applying Fridericia's formula was relevant for comparing action potential and calcium handling dynamics across variable beat rates for different tissues, field pacing was used for certain tissues at day 15. 1–3 Hz field pacing was applied using a Myo-pacer EP instrument (IonOptics). Two electrodes were positioned into the wells so that the applied voltage traveled through the individual tissues. 30 V, 20 ms bipolar pulse trains were applied. This formula was found to accurately convert the measurements from up to 3 Hz in 0 mm tissues, which was deemed appropriate for our data as no tissues spontaneously beat above approximately 3 Hz [Figs. S3.2(b)]. Although there was some discrepancy between the beat-rate corrected values for the 1- and 2-mm tissues at higher pacing frequencies, there were no elongated tissues that had a spontaneous beat rate above 1.5 Hz [Fig. S4(c)]. The majority of these tissues beat in a time frame in which Fridericia's formula minimally changes the quantified values.

### Statistical Analysis

GraphPad Prism 8.3.1 was used for statistical analysis. Ordinary one-way ANOVA was used for comparing multiple data groups before performing post-hoc Holm-Sidak mean comparison test. *P* value < 0.05 was considered statistically significant difference. ^*^ = p < 0.05; ^**^ = p < 0.005; ^***^ = p < 0.0005. Non-linear curve fitting and determination of EC_50_ were performed using a 3-parameter fit.

## SUPPLEMENTARY MATERIAL

See the supplementary material for additional information on methods including COMSOL modeling of the prestress within the 3 *μ*HM, cell length and width quantification, and using Fridericia's formula to beat rate correct electrophysiology quantification. Also included are additional graphs of action potential and calcium transient parameters for WT and PKP2^−/−^ tissue both at baseline and after applying pharmacology to block the ion channels described in the manuscript. Images of western blots and graphs of their quantification are also included.

## Data Availability

The data that support the findings of this study are available within the article and its supplementary material.
